# Rupture of the Œsophagus

**Published:** 1849-10

**Authors:** C. Colegrove

**Affiliations:** Sardinia


### ART. IV.—*Rupture of the Œsophagus.*

To the Editor of the Buffalo Medical Journal.

Misal Ballard, aet. 30, of leuco-phlegmatic temperament, and delicate constitution, on the afternoon of Thursday, Sept. 13, accidently swallowed the pit of a red plum, (*prunus Americanus,*) which got arrested in the preternaturally contracted calibre of the œsophagus, causing intense pain and considerable dyspnœa. At the age of three years, he unfortunately swallowed a solution of caustic potash, from the effects of which he very narrowly escaped with his life. The œsophagus, or its mucous membrane, was supposed to have been much injured, and on cicatrization, to have become greatly constricted, as he ever subsequently suffered from dysphagia, and occasionally from the lodgement of some substance, at a point evidently closely proximate to the stomach. The amount of contraction may be estimated from the circumstance of his inability to propel, by the natural action of the muscular tube, a kernel of corn into the stomach. In consequence of this liability to obstruction of the canal, he kept within reach, a probang, nearly half an inch in diameter, and which he now used to dislodge, by forcible propulsion, the arrested pit. Immediately on swallowing the stone, he threw himself horizontally on the ground, and endeavored to eject it, by efforts at vomiting.

Previous to my arrival, he had employed the probang, three times, at short intervals, in the abortive and dangerous effort to drive the stone into the stomach. These efforts, as he told me, were forcible, and the second was followed by a jet of blood. Alarmed at the imminently perilous aspect of the case, and unwilling to assume the responsibility of directing any measure, which must, in all probability, aggravate the difficulty, and expedite an untoward result, I despatched a messenger for farther medical aid. But incited by the spur of extreme agony, and in opposition to my advice, the patient repeated the use of the probang—each time, evidently, with more hopeless cer ainty, impacting the stone in the substance of the œsophagus, until at last it suddenly retreated, and the patient exclaimed, "I believe it is down."

Not then fully appreciating the extreme minuteness of the calibre at the site of the arrested substance, I was willing to entertain a hope that it had gone in the right direction; forlorn, indeed, as no abatement of the pain followed. The site of the stone, as impacted, was the nucleus of pain, which radiated upwards to the shoulders, and to the right side. Patient desired to drink, and was permitted to swallow a little cold water, which he said, "felt as if it went into the stomach." There was a troublesome accumulation of mucus in the throat, which he lacked strength to bring up, and which aggravated the sense of suffocation. Pulse, 60 per minute, and compressible; extremities cold, with copious perspiration from the neck and face. Administered a weak mixture of brandy and water, followed by a small dose of morphine. Directed a sinapism to the epigastrium. In a short time pulse rose to 100, then 120 — a rate maintained all night, when it reached 140. He was allowed (perhaps injudiciously,) to drink at intervals, till within a few hours prior to dissolution.

*Friday Morning.* — A tumefaction began to appear above the right clavicle, which constantly increased, and distinctly emphysematous. Respiration laborious, performed nearly exclusively by the diaphragm. In my absence, the attendants administered a dose of oil. Hands obstinately cold, perspiration profuse and clammy, hippocratic countenance. During the night I had prognosed inevitable death. To this consummation all the symptoms steadily pointed, until about five o'clock Friday afternoon, when the patient revived a little, and in a husky, inarticulate voice, expressed himself easier. Directed an enema of salts and senna. No perceptible alteration ensued until about 12 o'clock, when he sunk into insensibility, and expired at sunrise, 38 hours after the accident.

*Post Mortem.*—The lungs were found adherent, in many places, to the walls of the chest, and we detected a small quantity of pus issuing from some of the bronchial ramifications. The right lobe of the liver was considerably enlarged, and slightly altered in texture. The stomach was empty, and void of the minutest trace of disease. We removed more than a quart of reddish fluid from the cavity of the chest, which had unquestionably leaked through an aperture in the œsophagus, slight hemorrhage from the wound having given it a fleshy tinge. Upon a little manipulation, we discovered the unnatural orifice through which the stone had been propelled, distant nearly three inches from the cardiac orifice. The stone itself, having perforated the right wall of the tube, rested obliquely in the cellular investive of the œsophagus, two inches below the point of rupture. We noticed considerable sanguineous infiltration, along the anormal track of the foreign body. We did not succeed in detecting any rupture of the mediastinum or lungs, which might not have been occasioned by the manœuvre of manipulation. Intestines excessively tympanitic. The entire calibre of the œsophagus was exceedingly small, and at the narrowest point not larger than a pipe-stem.

Could this man have been saved? is the first question that arises on a review of the whole case. In my opinion, a negative answer is almost inevitable. Had the patient refrained from all effort to force the foreign body downwards—itself must have occluded the canal, so as to impede the passage of medicine, and every effort at deglutition would obviously more obstinately impact it in its anormal site. Evidently, emesis was the only rational mode that could have been suggested for accomplishing its removal. But even, in the event of its ejection, the delicate œsophageal membrane might have sustained fatal lesion. But after a third introduction of the probang, and the exercise of propulsive force, equivalent, as the patient thought, to several pounds, (though this is hardly probable,) what but the most desperate heroism could have expected to compass the danger, and disengage the stone by extraction? Indeed, the whole tube was quite too narrow to admit of but an imperfect and embarrassed use of forceps. The point of impaction was considerably remote. In efforts at extraction increased laceration would be all but inevitable. Seizure would have been extremely difficult, from the peculiar shape and surface of the body. The stone of this kind of plum is unusually flat, and its edges correspondingly sharp. There are other suggestions connected with the subject, but I forbear. Emphysema, I may add, is commonly attributed to rupture of the lungs. But I do not think there was any pulmonary lesion in this instance. M. Louis avers that it may happen, independently of any rupture of the air cells. I believe there are recorded, numerous instances of what has been termed spontaneous emphysema. In the present case, there is undoubted reason to suppose the mediastinum was wounded. I will simply append a paragraph from Dorsey's Cooper:

"We may infer that from a mere rupture of a few of the bronchial cells, occasioned by irregular action of the lungs, or some other internal cause, a spontaneous diffusion of air may take place in the cellular texture of the body. Such examples are dependent on the same cause as the emphysema, from injury of the lungs; only the rupture of the bronchial cells in the former cases is less obvious."

Yours, truly, C. COLEGROVE.

Sardinia, September 18, 1849.

